# Molecular characterization of Umbre virus (Bunyaviridae)

**DOI:** 10.1186/1743-422X-5-115

**Published:** 2008-10-08

**Authors:** Pragya D Yadav, Akhilesh C Mishra, Devendra T Mourya

**Affiliations:** 1 Microbial Containment Complex, National Institute of Virology, 130/1 Sus Road, Pashan, Pune 411021, India

## Abstract

Umbre (UMB) virus was first isolated from India in 1955 and classified as Orthobunyavirus (Turlock serogroup). Eight isolates of this virus, isolated from Culex mosquitoes were characterized on the basis of partial glycoprotein (G2) gene. Twenty-six percent differences at nucleotide level while 17% differences at amino acid level were noted within different isolates. Phylogentic data shows that this virus represents a distinct group within the genus Orthobunyavirus.

## Findings

The viruses of *Bunyaviridae *family are spherical particles, range 80 to 120 nm in diameter and share a common genetic organization of three predominantly negative stranded RNA segments (S, M and L). Based on antigenic, genetic and ecological relatedness, the Bunyaviruses are divided into five genera. The genus Orthobunyavirus includes approximately 60 viruses, which are known to cause disease in humans (Elliot, 1996). Virological surveillance of these viruses depends primarily on detecting the viruses in arthropod vector populations in nature. Although, serological test like immunoassays are available for antigen detection for a few viruses, cross-reaction in closely related viruses cannot be ignored (Artsob *et. al*., 1984; Hildreth *et. al*., 1982).

Umbre virus (UMB) [strain G-1424] was first isolated from *Culex bitaeniorhynchus *mosquitoes, collected in 1955 at Umbre, Kolaba district, Maharashtra State, India. The virus has been registered in the International Arbovirus Catalogue No. 43 (Dandwate *et. al*., 1969). During further field investigations, seven more strains of virus were isolated from *Cx. vishnui *mosquitoes. Recent reports on Bunyaviruses via. Ganjam virus isolation from Maharashtra (Joshi *et. al*., 2004) and antibody detection of Hantan virus in India (Chandi *et. al*., 2005), has provided evidences that Bunyaviruses are circulating in this country but their involvement in causing human and animal disease are not known yet. In the Gene Bank, only one sequence of Turlock serogroup i.e. N gene of M'poke virus is available.

UMB viruses used in this study are listed in (Table 1) along with their geographical origin, host source and year of isolation. The available eight strains of this virus was procured from the virus registry of National Institute of Virology, Pune and propagated in *VeroE-6* cells. Cytopathic effect (CPE) was observed during 4^th ^– 6^th ^post infection day. Infected cells were harvested, centrifuged and supernatant was used for molecular characterization of the virus.

**Table 1:** Details of the virus strains used in the current study

Strain no.	Year of isolation	Host association	Place of isolation	Accession No.
G-1424	1955	*Culex bitaeniorynchus*	Umbre, Maharashtra	**EU697948**
G-7441	1956	*Cx. vishnui*	Kammavanpet, Tamil Nadu	**EU697945**
G-8335	1956	*Cx. vishnui*	Minnal, Tamil Nadu	**EU697946**
G-9601	1956	*Cx. vishnui*	Sulari, Tamil Nadu	**EU697947**
G-16283	1957	*Cx. vishnui*	Sathuperi, Tamil Nadu	**EU678356**
G-16310	1957	*Cx. vishnui*	Sathuperi, Tamil Nadu	**EU697942**
631308	1963	*Cx. vishnui*	Vellore, Tamil Nadu	**EU697944**
809365	1980	*Cx. vishnui*	Muduvadi, Karnataka	**EU697943**

RNAs were isolated using chloroform, isoamylalcohol and further purified using RNAaid kit (Biogene), according to the manufacturer's instructions. RNAs were dissolved in 50 μl nuclease free water. Different sets of primers were used to amplify partial N, L and M gene. Partial M gene of 570 bp could be amplified using primer pair M14C and M619R, as described by Bowen *et. al*., (2001), represents the nucleotide sequences of the N-terminal half of the G2 glycoprotein. Superscript III single step RT-PCR with Platinum Taq DNA polymerase kit (Invitrogen) was used for amplification of partial M gene according to the manufacturer's instructions.

Amplified products were detected in 2% agarose gel after staining with ethidium bromide in Tris/acetate/EDTA buffer (TAE). A desired size of 575 bp product was purified using QIAquick gel extraction kit (Qiagen), as per manufacturer's instructions. The sequences of amplified products were determined by using ABI PRISM BigDye Terminator V3.1 cycle sequencing ready reaction kit (Applied Biosystems). Amplification primers were used to sequence the amplified products. Cycle sequencing PCR program was used for 96°C-1 min, 96°C-10 sec, 50°C-5 sec and extension of 2 min at 60°C for 30 cycles.

The partial M gene sequence was curetted with the help of KODON Software and aligned with known Gene Bank sequences of Bunyamwera serogroup, California serogroup, and Kaeng Khoi viruses using clastal *W *program. Phylogenetic analysis was performed using Mega 3.0 by using neighbor-joining algorithm with thousand bootstrap values.

**Figure 1:**
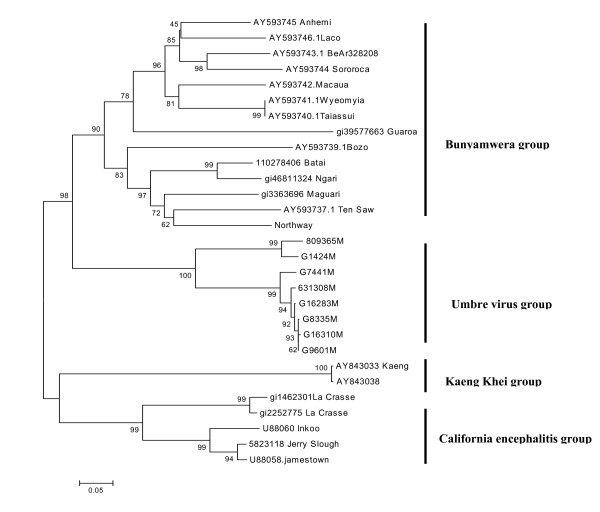
**Phylogenetic comparison of partial M RNA segments of the Umbre virus with other Orthobuyaviruses**. Using Mega 3.0 software Neighbor-joining analysis performed with 1000 bootstrap replicates. Umbre virus forms a separate group within *Orthobunyaviruses*. Partial M segment source are: Umbre virus strain no G-16310 (EU697942), 809365 (EU697943), 631308 (EU697944), G-7441 (EU697945), G-8335 (EU697946), G-9601 (EU697947), G-1424 (EU697948) and G-16283 (EU678356).

Partial M gene sequences showed maximum homology with Bunyamwera serogroup virus. Nucleotide and amino acid similarity within eight isolates varied from 74–100% and 83–99% respectively. Isolate G1424 and 809365 come together with 6% and 1% difference of nucleotide and amino acid respectively, while other six isolates club together with 5% nucleotide and 4% amino acid differences (Figure 1). Nucleotide and amino acid homology in UMB viruses ranged from 49–75% and 25–84% respectively with other Orthobunyaviruses. There were 26 amino acid conserved sites as compared to other Orthobunyaviruses.

UMB virus was isolated from two different species of Culex mosquitoes i.e. *Cx. bitaeniorhynchus *and *Cx. vishnui *which were collected from three different states. Both the species have different host range and breeding habitats, but the viruses obtained from these mosquito species were not different on the genomic bases. The first UMB isolate [strain no. G 1424] and the last isolate [strain no. 809365] from India showed maximum similarity while other six isolates club together. Phylogenetic analysis of genetically characterized member of the genus Orthobunya (345 bp of glycoprotein gene G2, data not shown) are divides into six distinct lineage viz. Bunyamwera, Simbu, California encephalitis, Group C, Kaeng Khoi (KK) and UMB virus. Comparison of 550 bp of partial M gene showed four lineages, excluding Group C viruses and Simbu group of virus (Figure 1). The genetic divergence between these lineages suggests that UMB virus represent a distinct group within the genus *Orthobunya*.

UMB virus strain G 1424 and 809365 not only highly homologues on the genomic level but also in their cell infectivity pattern. These two strains took more time to show CPE in comparison of other six isolates. Complete genome sequencing may shed light, why these two isolates are separate from other six, which club together despite isolated from two different mosquito species.

Turlock and Umbre virus are distinct from each other based on neutralization test (Calisher *et al*., 1984). Availability of more sequences of Turlock group may answer about placement of this group of viruses. Bunyaviruses being three segmented RNA viruses have the capacity to reassort their segments into new genetically distinct viruses, if the target cells are subject to dual infection. The possibility of drift, shift and UMB virus evolution towards an emerging disease pathogen cannot be predicted based on partial sequences. Complete genome sequencing of UMB virus can possibility suggest whether there is any reassortment between three genes of this virus as known for Ngeri, Batai and Jatobal virus (Briese *et. al*., 2006; Yanase *et. al*., 2006; Saeed *et. al*., 2001).

## Competing interests

The authors declare that they have no competing interests.

## Authors' contributions

PDY performed the PCR and sequencing. ACM helped in preparation of manuscript. DTM and PDY designed, coordinated the study and prepared the manuscript.
